# Mechanism based approaches for rescuing and enhancing cognition

**DOI:** 10.3389/fnins.2013.00143

**Published:** 2013-08-15

**Authors:** Gary Lynch, Christine M. Gall

**Affiliations:** ^1^Department of Psychiatry and Human Behavior, University of CaliforniaIrvine, CA, USA; ^2^Department of Anatomy and Neurobiology, University of CaliforniaIrvine, CA, USA; ^3^Department of Neurobiology and Behavior, University of CaliforniaIrvine, CA, USA

**Keywords:** long-term potentiation, LTP, ampakine, learning, cognitive enhancement, spaced trials, animal models, cytoskeleton

## Abstract

Progress toward pharmacological means for enhancing memory and cognition has been retarded by the widely discussed failure of behavioral studies in animals to predict human outcomes. As a result, a number of groups have targeted cognition-related neurobiological mechanisms in animal models, with the assumption that these basic processes are highly conserved across mammals. Here we survey one such approach that begins with a form of synaptic plasticity intimately related to memory encoding in animals and likely operative in humans. An initial section will describe a detailed hypothesis concerning the signaling and structural events (a “substrate map”) that convert learning associated patterns of afferent activity into extremely stable increases in fast, excitatory transmission. We next describe results suggesting that all instances of intellectual impairment so far tested in rodent models involve a common endpoint failure in the substrate map. This will be followed by a clinically plausible proposal for obviating the ultimate defect in these models. We then take up the question of whether it is reasonable to expect, from either general principles or a very limited set of experimental results, that enhancing memory will expand the cognitive capabilities of high functioning brains. The final section makes several suggestions about how to improve translation of behavioral results from animals to humans. Collectively, the material covered here points to the following: (1) enhancement, in the sense of rescue, is not an unrealistic possibility for a broad array of neuropsychiatric disorders; (2) serendipity aside, developing means for improving memory in normals will likely require integration of information about mechanisms with new behavioral testing strategies; (3) a shift in emphasis from synapses to networks is a next, logical step in the evolution of the cognition enhancement field.

## Introduction

The term “cognitive enhancement” often refers to attempts at reducing the intellectual impairments associated with a very broad array of psychiatric disorders. Recently, however, there has been considerable discussion about the possibility of extending the concept to include effects in high functioning individuals (Turner et al., 2003; Cakic, 2009; Farah et al., 2009; Hyman, 2011). Questions are being asked not only about feasibility but also about the ethics and societal consequences of developing agents that expand human mental capabilities (Darby, 2010; Sahakian and Morein-Zamir, 2011; Forlini et al., 2013). This surprising surge of interest likely reflects increased understanding of the synaptic mechanisms that encode memory, and new information on how these processes might be disturbed in animal models of various psychiatric disorders. Notably, work in this field has led to a number of therapeutic strategies, some of which have progressed to clinical trials. Success in any of these efforts could result in treatments for persons with congenital or emergent impairments as well as for potential “off label” use in normals.

In addition to stimulating interest in cognitive enhancement, the advent of plausible, plasticity-based therapeutics could prove to be an important conceptual step in the evolution of the field. Past work largely involved compounds developed for uses not directly related to memory encoding events but found to improve learning in animals. The newer approaches target specific links in the chain of cell biological events leading to persistent synaptic changes, and thus have the potential to produce agents with relatively selective effects. This specificity could prove to be critical for translation. The success of neurobiologically grounded strategies does, however, depend on the validity and richness of current hypotheses about the nature of memory substrates, a topic that continues to be the subject of some controversy. The present review will describe a detailed model, much of which is derived from work using recently introduced imaging techniques, of the events responsible for the stabilization of long-term potentiation (LTP) at forebrain synapses in adult rodents. It will be argued that this “substrate map” explains a great deal of well-documented LTP phenomenology and as well-provides a new set of timing rules logically related to a fundamental characteristic of memory largely ignored by neuroscientists. Subsequent sections will consider the utility of the working hypothesis in identifying critical defects in rodent models for a variety of human conditions in which memory problems are prominent. Regarding this, we will use the map to suggest that the animal models so far tested all have a common endpoint failure in the cytoskeletal machinery that stabilizes memory. This conclusion, were it to be correct, would raise the possibility of broad spectrum cognitive enhancers that rescue memory, and perhaps cognition, across many different instances of intellectual disability. Tests of this idea will be described using a particular class of drugs.

The review next takes up the question of whether treatments that rescue memory in rodent models produce cognitive enhancement in normals. This will incorporate a discussion of why modifying synaptic characteristics might produce network level effects that lead to greater cognitive capabilities. Finally, a critical issue in contemporary work on enhancement concerns the reasons why results obtained with animal models have a poor record of predicting human outcomes. We will consider this essential question at various points in the review, beginning with the following section.

## The problem of predicting cognitive effects in humans from animal results

**Figure 1:**
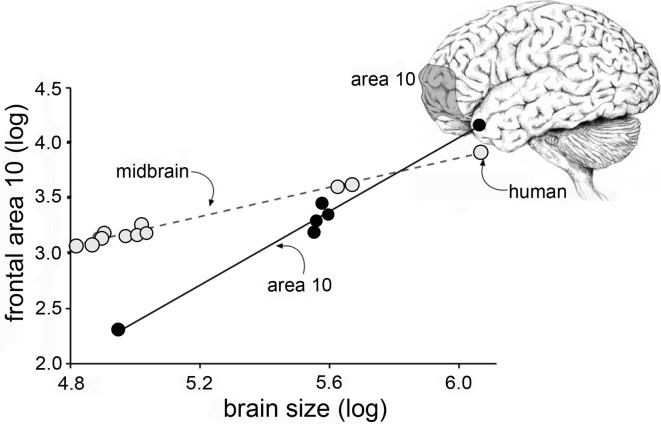
**Cortical areas expand disproportionately with increases in brain size.** Plot shows the relative size of the midbrain (monkeys, apes, human) and frontal cortical area 10 (apes and human, only) plotted as a function of overall brain size. As shown, the slope for midbrain is relatively flat: with a slope of <1 the midbrain occupies a progressively smaller proportion of brain volume as brain sizes increase. In contrast, with a slope of >1, Area 10 grows disproportionately to overall volume with increases in brain volume (Area 10 results from Semendeferi et al., 2001).

Research on cellular processes related to cognition necessarily depends on animal, and primarily rodent, studies that face formidable hurdles with regard to human relevance. For one, human brains have very different proportions than those of rodents. Rats are small mammals with expectedly small brains while humans are among the larger members of the class and have disproportionately (about 3-fold relative to body weight) large brains. Differences in brain size are associated with changes in the proportions of brain regions according to surprisingly precise allometric rules that apply across the several orders of mammals (Lynch and Granger, 2008, for a review). Thus, where cortex constitutes about 30% of the rat brain, the corresponding figure for humans is 80%. And, making matters worse for translation, the cortical expansion leads to changes in the relative sizes of specific cortical fields. For example, Area 10, a region intimately involved in cognitive processing, occupies a much greater percentage of cortex in man than it does in other primates (Figure 1) (Semendeferi et al., 2001). These general points have long been recognized by neuroanatomists and incorporated into a broad hypothesis referred to as “encephalization of function.” This idea, which appears to have fallen out of favor, posits that many functions become progressively more dependent on cortex than on lower structures as brain size increases. Fulton, in 1941, described several examples of how discrete cortical lesions produce increasingly profound and permanent impairments moving from carnivores to apes, and then to humans (Fulton, 1941). There is every possibility that the encephalization effect applies to performance in widely used learning and memory tasks; if so, treatments that enhance performance in rodents could be acting on brain structures other than those used by humans to deal with similar problems.

The above points may account for the much discussed failure of preclinical studies on memory enhancement to predict human outcomes (Davis et al., 2008; van der Worp et al., 2010; Menache, 2012). A long list of compounds improve retention scores in rodents and, to a lesser extent, monkeys but there are no approved treatments for humans. In some cases the failure of translation can be ascribed to safety questions but this is not the issue for drugs or everyday compounds in wide use for established indications. The great disparity between preclinical and clinical results has prompted neuropsychologists to develop behavioral paradigms that depend on complex computations that are both formally similar to those used by humans and associated with specific cortical structures, in particular hippocampus and frontal lobe (Demeter et al., 2008; Sauvage et al., 2008; Sarter et al., 2009; Sauvage, 2010; Lynch et al., 2011). These new behavioral assays substantially raise the bar for concluding that a given experimental treatment enhances human-like learning in animals; it is exciting that a small number of clinically plausible drugs appear to pass these challenging assays (Sarter et al., 2009).

The ongoing development of sophisticated behavioral technologies for sampling human-like learning in rodents brings to the forefront the question of what we mean by cognitive enhancement. It is probably fair to say that most, although certainly not all, of the work in this field makes the assumption that improving the speed of learning will result in improved cognitive functioning. This seems reasonable when considering intellectual disabilities but it is not at all clear that the argument will hold for high functioning individuals. Humans and other animals incorporate new memory into pre-existing psychological structures built upon extensive experience with complex environments, and thereby focus on relevant information while avoiding what is essentially noise (In these instances, “noise” is defined as stimuli which vary without consistent relationship to task execution). It follows from this that simply increasing responsivity of neural substrates of encoding could have as many negative as positive consequences for subsequent cognitive operations.

The development of animal tests that relate closely to humans and are based on homologous cortical regions, while critical to the future of the enhancement field, raises another and little explored question about cognition: how much of everyday cognitive performance is captured by the supervised, forms of learning used in these new tests. One of the great and long standing questions of experimental psychology revolves around the question of whether operant learning rules can explain the rapid, mastering of novel, complex environments. The problem is of immediate relevance to cognitive enhancement because it is very likely that much of human cognition involves unsupervised interactions of this type.

In all, improved animal behavioral protocols will provide far more challenging preclinical screens for cognitive enhancers than more commonly used tests. This should greatly improve success in predicting human efficacy in studies targeting either cognitive disorders or normal mentation. How well any improvements in problem solving extend to cognition in the great majority of real world circumstances is a fascinating follow-on question of great pragmatic and theoretical interest.

## Using presumably conserved neurobiological substrates as measures in the development of cognitive enhancers

A great deal has been learned about the synaptic events that lead to the expression and subsequent stabilization of a form of synaptic plasticity (LTP) that, from a very large number of studies using very different experimental approaches, appears to be critical to the encoding of memory (Bliss and Collingridge, 1993; Morris, 2003; Lynch, 2004b; Lynch et al., 2008, 2010; Sacktor, 2008). Much of the pertinent synaptic machinery is found throughout the cortical telencephalon in rodents and primates, including humans, and it is thus reasonable to assume that LTP-like effects occur in many regions critical to cognition. For example, LTP in rodents is commonly induced with a specific pattern of afferent stimulation (theta burst stimulation: TBS) (Larson and Lynch, 1986; Larson et al., 1986) that is also effective in promoting learning in human studies using transcranial magnetic stimulation (Ackerley et al., 2010; Hsu et al., 2011; Huang et al., 2011). These observations point to the possibility of using LTP, and the neurobiological mechanisms responsible for it, as a screen for evaluating enhancement strategies. Thus, while learning may be differentially dependent on cortical structures in rodents and humans, it may nonetheless involve the same neurobiological substrates that have similar cross-species reactions to experimental treatments. A great advantage of this approach with regard to evaluations of cognitive enhancement in cases of learning disorders is that it lends itself to a two-part strategy in which cellular defects are first identified in rodent models and then, using the same models, potential therapeutics are tested for their ability to correct or obviate those defects. A potential drawback to substrate-directed experiments is that they assume that improving learning will either rescue or enhance cognition, something that is not at all certain.

In this section, we will summarize a model of how LTP is consolidated. Subsequent portions of the review will then use this information to describe one implementation of the two-part strategy mentioned above. We would note that the model, though incorporating results from many research groups, depends heavily on work from our laboratories; quite different and intriguing hypotheses have been advanced, most notably by Sacktor and colleagues (Sacktor, 2008).

### Characteristics and substrates of LTP

**Figure 2:**
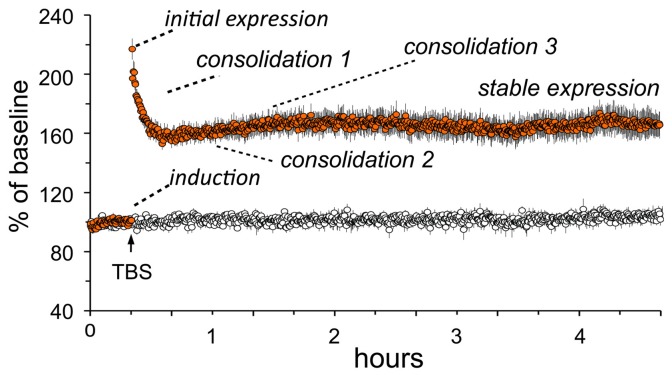
**Characteristics of long-term potentiation (LTP).** Stimulation was applied to two populations of Schaffer-commissural afferents converging on the stratum radiatum of field CA1b. Theta burst stimulation (TBS) delivered to one input (filled circles) causes an immediate increase in the size of the field EPSP which then decays over about 10 min to a plateau at which the responses are 50–60% elevated above the pre-TBS baseline. The potentiation persists unchanged for the duration of the recording session (nearly 5 h in the illustrated case). Note that the second (control) input, which received only 3/min stimulation pulses, was not affected by potentiation of neighboring contacts (open circles). Thus, LTP has the synapse specificity expected for a memory substrate. As with memory encoding, LTP is initially unstable and readily erased by a number of treatments (e.g., 5 Hz stimulation) but then becomes steadily more resistant to disruption. Experimental work indicates that this process involves multiple stages. A rapid phase (“consolidation 1”) has been linked to reorganization of the sub-synaptic cytoskeleton over the 10 min following TBS; this is followed at about 1 h by a newly discovered stabilization event (“consolidation 2”) involving synaptic adhesion receptors. There is also considerable evidence for a still later step that depends on protein synthesis (“consolidation 3”).

Essential features of adult LTP, as studied in acute hippocampal slices, are summarized in Figure 2. Note that theta burst stimulation (TBS; Larson and Lynch, 1986), which mimics patterns of neuronal firing that occur during learning (Otto et al., 1991; Buzsaki, 2005; Axmacher et al., 2006), involves very little stimulation (20–40 pulses) and yet leads to significant and enduring facilitation of EPSPs evoked by subsequent single stimulation pulses. The potentiation effect thus satisfies the rapid induction requirement of a memory mechanism. LTP is synapse-specific (see “control input” to the same dendritic zone in Figure 2) as required by the enormous capacity of memory. Finally, LTP is astonishingly persistent, an observation that is only hinted at by the 5 h slice recordings shown in the figure; LTP has been shown to last for months using *in vivo* recordings (Staubli and Lynch, 1987; Abraham, 2003). It therefore satisfies a third, particularly demanding requirement for a memory substrate: extreme stability.

**Figure 3:**
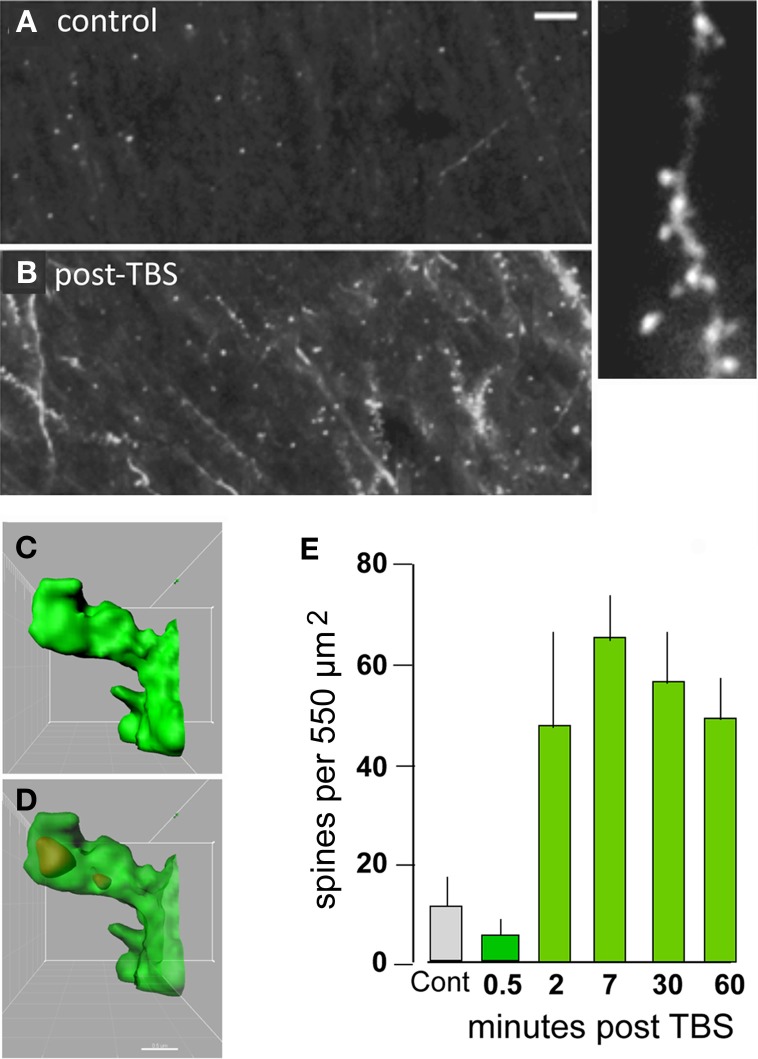
**Theta burst afferent stimulation (TBS) causes actin polymerization in dendritic spines.** Fluorescent-tagged phalloidin was infused into living hippocampal slices to label filamentous (F) actin; after harvest the slices were sectioned for epifluorescence microscopy. **(A,B)** Images at left show that spine-labeling (white puncta) is very low in a control slice but prominent following TBS. At higher magnification (at right) labeled puncta can be seen to be spines decorating a faintly labeled dendrite. **(C,D)** Combining phalloidin labeling with green fluorescent protein expression allows confocal visualization of phalloidin-labeled puncta within the heads of clearly defined spines; panels show opaque 3D build of a single spine **(C)** and a semi-transparent rendering showing the phalloidin-labeled aggregate within the spine's boundaries **(D)**. **(E)** Quantification of densely phalloidin-labeled spines at different time points following TBS shows that numbers are significantly increased, relative to values in control (cont.) slices, as early as 2 min post-TBS and remain elevated through 60 min after stimulation (*p* > 0.001 for 2–60 min vs. cont). Modified from Kramar et al. (2012b).

What types of cellular events could produce such exotic, with regard to duration, physiological effects? Some time ago we proposed that expansion of the synaptic region, due to reorganization of the actin cytoskeleton, results in an increase in the size of the post-synaptic receptor pool and thus to enhanced transmission (Lynch and Baudry, 1988). Direct tests of the idea that TBS triggers the formation of subsynaptic actin networks became possible with the development of methods that selectively label actin polymers in living hippocampal slices following induction of LTP (Lin et al., 2005; Kramar et al., 2006). Studies using this method confirmed that TBS causes a marked, NMDA receptor-dependent increase in the number of spines containing high concentrations of filamentous (F-) actin (Figure 3) and that this effect lasts for at least 90 min (the longest time tested) (Kramar et al., 2006; Rex et al., 2009, 2010). It was known from previous work that toxins which block actin filament assembly prevent LTP consolidation (Krucker et al., 2000), a point that we have confirmed using multiple interventions (see below).

### Linking theta burst stimulation to LTP-related cytoskeletal reorganization

**Figure 4:**
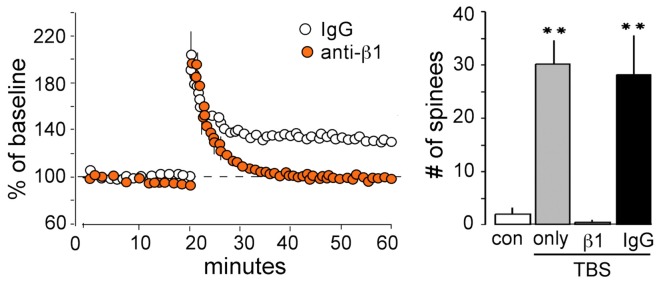
**ß1 integrins are required for TBS-induced LTP and increases in spine F-actin. Left**: Plot shows that potentiation of the Schaffer-commissural projection to field CA1 is induced with TBS applied in the presence of the control anti-rat IgG but is blocked with local infusion of neutralizing antisera to ß1 integrin: note that with ß1 neutralization there is an initial post-TBS potentiation but the enhanced response rapidly declines to baseline indicating a failure in consolidation. **Right**: Quantification of spines containing dense F-actin in the CA1 field of afferent stimulation (from *in situ* phalloidin labeling). As shown, TBS elicits a large increase in spine F-actin if applied alone or in the presence of anti-rat IgG but this effect is totally blocked by neutralizing anti-ß1 (^**^p < 0.001).

**Figure 5:**
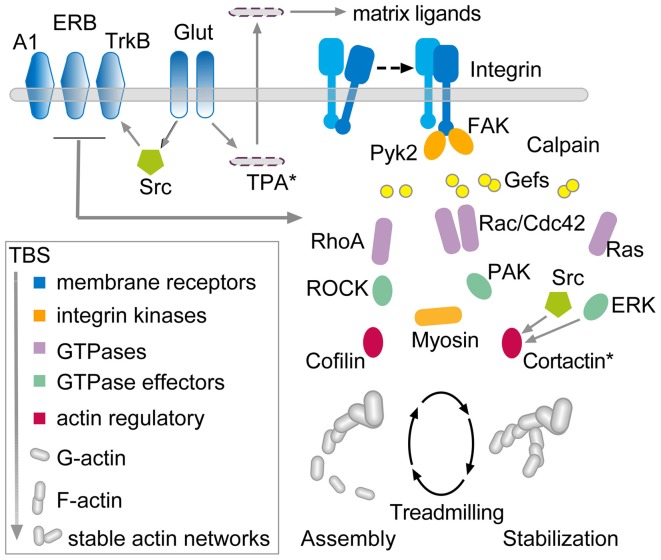
**Hypothesis regarding the links between theta burst stimulation and the cytoskeletal changes underlying rapid consolidation of LTP.** The model uses three groups of transmembrane receptors (in blue) for the following: (1) “modifiers” including adenosine (A1), estrogen (ERB), and BDNF (TrkB); (2) released neurotransmitter (Glut); and (3) adhesion proteins (integrins). The last of these, working in conjunction with the modifier group, signal through guanine exchange factors (Gefs) to the small GTPases (violet) which, in turn, activate downstream intermediaries (green) leading to actin regulatory proteins (red) that ultimately control the activity-driven assembly and subsequent stabilization of actin filaments. There is evidence that the RhoA-ROCK-Cofilin path controls F-actin assembly whereas Rac and Ras signaling, including convergence on cortactin, is thought to mediate the stabilization and elaboration of the actin network.

Transmembrane receptors that bind extracellular matrix proteins (integrins) exert potent effects on the organization of the submembrane cytoskeleton at adhesion junctions throughout the body. Given that synapses are the principle adhesion junctions in brain, integrins were viewed as likely mediators of the striking increases in spine F-actin produced by TBS. This idea was all the more attractive because of studies using integrin blocking with the ligand mimetic peptides, toxins, and genetic manipulations had shown that disruption of integrins thoroughly blocks hippocampal LTP (Staubli et al., 1990, 1998; Xiao et al., 1991; Chun et al., 2001; Kramar et al., 2002, 2006; Chan et al., 2003; Huang et al., 2006; Nagy et al., 2006; Bozdagi et al., 2007) and the formation of long-term memory (Chan et al., 2003, 2006, 2007; Nagy et al., 2007; Babayan et al., 2012). Direct confirmation that integrins play a critical role in the formation of actin polymers associated with LTP induction came with studies using infusions of neutralizing antisera against ß1 integrins, the subtype of adhesion receptors found in hippocampal synapses. Those experiments showed that blocking ß1-family integrins eliminates both spine actin polymerization and LTP consolidation (Kramar et al., 2006) (Figure 4). The question then became one of how the assumed integrin activation by TBS triggered the assembly and stabilization of actin filaments. Research from cell biologists has described a number of likely pathways (Miranti and Brugge, 2002; Brakebusch and Fassler, 2003; DeMali et al., 2003; Danen et al., 2005; Wiesner et al., 2005; Warren et al., 2012) but testing for these with regard to LTP is difficult because the potentiation effect occurs in the small percentage of the synaptic population affected by conventional stimulation paradigms (see Chen et al., 2007). Accordingly, we developed a dual immunofluorescence method that can be used to reconstruct a large number of the synapses (labeled with antibodies for the post-synaptic density protein PSD95) within the field of synaptic potentiation and so increases the likelihood of detecting, with a second state-dependent antibody, those contacts containing an activated (e.g., phosphorylated) variant of a given actin signaling protein. Using this Fluorescence Deconvolution Tomography (FDT) method, and various experimental manipulations (drugs, toxins, genetic) to target specific receptors and signaling intermediaries, we evaluated activities of actin management and LTP-related proteins that are concentrated at excitatory synapses. This effort resulted in the model linking transmembrane receptors to the dendritic spine actin cytoskeleton described in Figure 5.

The model is complex and there is not sufficient space here to discuss the individual studies that led to its construction. However, the arrangements can be broken into three components. First, three groups of membrane receptors—“modifier,” transmitter, and adhesion—respond directly or indirectly (via AMPA and NMDA receptors) to TBS. The neurotransmitter and adhesion groups initiate at least four signaling cascades involving various small GTPases, all of which are influenced by the modifier group. One of these cascades, consisting of RhoA, its effector ROCK, and ultimately the actin severing protein cofilin, triggers the formation of new actin polymers in less than 2 min following TBS. Experiments using various treatments suggest that the newly formed filaments are initially unstable and can be disassembled by, for example, low frequency afferent stimulation or adenosine infusion (Rex et al., 2009). The actin filaments become progressively more stable over 5–10 min at which point these same reversing treatments are no longer effective. We hypothesize that the remaining GTPase driven pathways (i.e., through Rac/Cdc42, Ras, and Raf) provide for this stabilization, an idea that has received some experimental support (Rex et al., 2009; Chen et al., 2010), and also serve to organize the new polymers into structured actin networks. The time frame for this cytoskeletal stabilization corresponds well with that for the rapid consolidation of LTP, a process first identified with the same treatments used to disrupt the newly formed filaments (Larson et al., 1993; Huang et al., 1999). Importantly, interrupting various steps in the model disrupts the transfer of newly learned material into long-term memory (Dash et al., 2004; Rex et al., 2010; Lamprecht, 2011; Babayan et al., 2012; Gavin et al., 2012).

The above collection of results describes well-defined targets for testing if a putative cognitive enhancer either lowers the threshold for inducing learning-related synaptic changes or increases the percentage of activated synapses that exhibit such effects after supra-threshold stimulation. The combination of physiological recording and evaluating activities in the multiple actin regulatory signaling cascades would yield quantitative measures of selectivity. As discussed next, they would also provide an organized system with which to search for potential causes of the learning problems associated with intellectual disabilities and so a test bed for evaluating the normalizing potential of candidate cognitive enhancers.

## A final common pathway for learning impairments?

**Figure 6:**
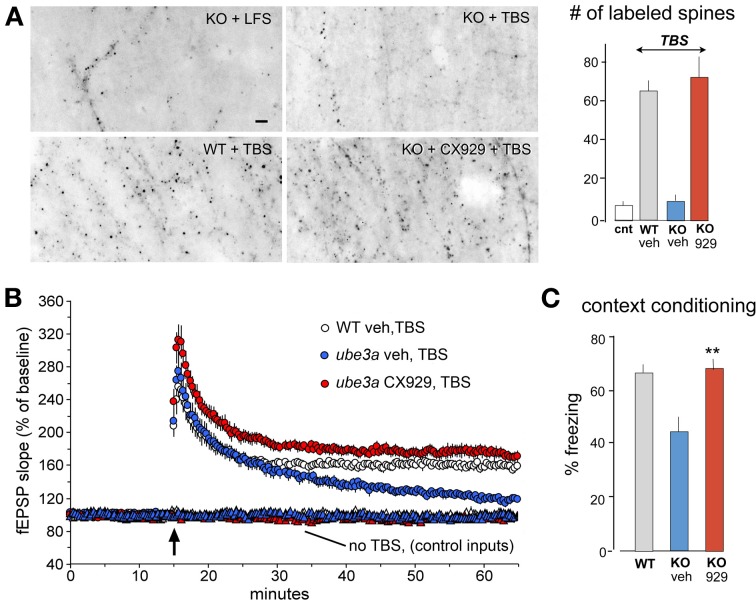
***In vivo* ampakine treatments rescue TBS-induced spine actin polymerization and LTP in a mouse model of Angelman syndrome.** Wild type (WT) mice and mutants with a knockout (KO) of the maternal *Ube3a* gene (AS mice) were treated with the ampakine CX929 or vehicle daily for 5 days prior to the preparation of acute hippocampal slices. **(A)** *In situ* phalloidin labeling was used to assess effects of genotype and treatment on the marked increase in spine F-actin that normally follows TBS. Images and quantification of F-actin rich (phalloidin-labeled) spines show that TBS does not increase the number of such spines in slices prepared from KOs treated with vehicle (veh) relative to counts from slices receiving control (cnt) low frequency stimulation (LFS) only. In contrast, the same stimulation applied to slices from KO mice previously treated with CX929 *in vivo* (lower right image) elicits a striking increase in F-actin rich spines that is comparable to that obtained in WTs (lower left image). **(B)** Plots of fEPSP responses show that TBS (arrow) elicits initial potentiation in all groups but the effect slowly decays back to baseline levels in vehicle-treated Ube3a mutants (*ube3a* veh). However, slices from mutants given CX929 *in vivo* (*ube3a* cx929) exhibit the conventional LTP effect. **(C)** Bar graph shows that context fear conditioning is also rescued in the KOs treated with CX929 (^**^p < 0.01 vs. KO veh). Adapted from Baudry et al. (2012).

Significant LTP impairments are present in animal models for an impressive list of human conditions associated with memory problems (Table 1). We have found defects in components of the neurobiological substrate model described in Figure 5 in each of the cases thus far studied. For example, TBS-induced actin polymerization is absent, and hippocampal LTP impaired, in mouse models of early stage Huntington Disease (three models) (Lynch et al., 2007; Simmons et al., 2009), Angelman syndrome (Baudry et al., 2012) (Figure 6), inflammation (Tong et al., 2012), and chronic depression of estrogen levels (Kramar et al., 2009a,b). The *Fmr1* KO model of Fragile X syndrome presented an interesting condition in which actin filaments formed after TBS but did not properly stabilize (Chen et al., 2010). The diverse causes of the target conditions points to an hypothesis in which failures in cytoskeletal reorganization initiated by learning-related patterns of afferent activity, and needed for memory formation, are a common neurobiological substrate for intellectual impairment in the different disorders. They also strongly suggest that the conditions have defects at different sites in the complex machinery leading to actin filament assembly and/or stabilization. Identifying where signaling fails will be of great utility in deciding how any cognitive enhancer that normalizes actin network formation, LTP, and learning produces such effects. The optimal outcome would presumably be that the therapeutic selectively restores a broken link in signaling. However, given the overlap in the pathways and thus likely redundancies, recovery of downstream events could reflect a circumvention of the defect. In considering these possibilities, we have used FDT and other methods to investigate signaling in a subset of the models.

**Table 1:** **Models of human intellectual disability have deficits in LTP and spine actin management**.

**Human condition**	**Model**	**Actin**	**Demonstration of**
			**LTP impairment**
Huntington Disease	Hdh^Q111^, Hdh^Q92^	P	Lynch et al., 2007
	CAG140	P	Simmons et al., 2009
	R6/2		Murray et al., 2000
Angelman syndrome	*Ue3a* mutant	P	Jiang et al., 1998; Baudry et al., 2012
Low estrogen levels	OVX rat	P	Kramar et al., 2009a,b, 2012a, 2013
Inflammation	IL-1ß treatment	P	Murray and Lynch, 1998; Tong et al., 2012
Fragile X syndrome	*Fmr1* KO	S	Lauterborn et al., 2007; Chen et al., 2010; Lee et al., 2011
Down syndrome	Ts65Dn		Costa and Grybko, 2005; Belichenko et al., 2007
Rett syndrome	*Mecp2* KO		Moretti et al., 2006
Tuberous sclerosis	Tsc2^+/−^		von der Brelie et al., 2006; Ehninger et al., 2008

All rodent models listed have impairments in the consolidation of hippocampal LTP. For all cases evaluated, disturbances in actin regulation are evident after theta burst afferent stimulation and involve either a failure in new actin polymerization (“P”) or in the normal stabilization of new actin filaments (“S”).

A fairly straightforward explanation can be offered for the loss of actin signaling and LTP in the case of the Huntington Disease models: the disease is multiply reported to reduce the concentrations and trafficking of Brain-Derived Neurotrophic Factor (BDNF) (Zuccato et al., 2001; del Toro et al., 2006), which acts on one of the “modifier” receptors (TrkB) described in Figure 5. Several groups, including our own, have shown that sequestering extracellular BDNF disrupts LTP consolidation (Kovalchuk et al., 2002; Rex et al., 2007); moreover, infusions of the factor stimulate RhoA signaling leading to actin filament assembly and LTP (Kramar et al., 2009b, 2013). Indeed, such treatments fully rescue actin polymerization and potentiation in Huntington Disease model mice (Lynch et al., 2007). Recent experimental work suggests that inflammation also blocks BDNF signaling but in this case because interleukin-1ß interferes with the cascade linking BDNF's TrkB receptor to downstream effectors, including cofilin (Tong et al., 2012). Low levels of estrogen, and thus under-stimulation of estrogen receptor beta, also results in a failure of TBS to engage RhoA signaling to cofilin (Kramar et al., 2009b). Somewhat surprisingly, acute treatments with the hormone completely rescue TBS-induced actin polymerization and LTP in middle-aged rats with long-term ovariectomies (Kramar et al., 2012a). Sustained stress, acting through the CRHR_1_ receptor for locally released Corticotrophin Releasing Hormone, excessively activates RhoA and thereby blocks new actin filament assembly with TBS (Chen et al., 2013). Normal aging in rat appears to increase extracellular levels of adenosine and thus cause overstimulation of the post-synaptic A1 receptors (Rex et al., 2005); this is a likely contributor to the loss of LTP in middle aged rats because A1 receptors negatively modulate TBS-induced RhoA to cofilin signaling and actin polymerization (Rex et al., 2009). The RhoA pathway appears to be intact in the *Fmr1* KO Fragile X syndrome model mouse (Lauterborn et al., 2007) but we have obtained evidence that TBS fails to engage other GTPases and downstream elements involved in the stabilization of cytoskeletal changes in these mice (Chen et al., 2010; Seese et al., 2012).

As expected, impairments to a final common LTP-essential event (reorganization of the actin cytoskeleton) in models for conditions with different etiologies are associated with defects at quite different upstream sites. But surprising generalities are present as well: excluding Fragile X, all of cases so far studied involve the RhoA pathway, and in two instances (inflammation, Huntington Disease) this reflects a problem with BDNF “transmission.” In line with our expectation that this will hold for other models in which TBS-induced actin polymerization is impaired, it was recently reported that synaptic BDNF signaling through TrkB is markedly attenuated in *Ube3a* KO (Angelman syndrome) mice (Cao et al., 2013). However, the results for *Fmr1* KOs indicate that disturbances in the other small GTPase initiated signaling pathways described in Figure 5 will likely be found as animal testing proceeds and we suggest that these will ultimately be linked to processes underlying F-actin stabilization.

Clearly, a great deal more work is needed to characterize signaling impairments in models discussed above and to evaluate the integrity of these systems in models for other forms of cognitive impairment (e.g., Alzheimer Disease, Down Syndrome). Nonetheless, the development of a substrate map for rapid consolidation has provided a framework for detecting specific links affected by a diverse array of conditions that disrupt the memory encoding component of cognition in humans. This constitutes the first step in the two-part strategy alluded to earlier. We now turn to the second part: using this information to screen putative enhancers.

## Effects of potential cognitive enhancers on actin signaling defects and LTP impairments in models of intellectual disability

Here we will illustrate the idea of using the substrate map for LTP to evaluate putative cognitive enhancers with regard to the rescue of memory-related plasticity. The agent to be tested is a member of the ampakine family, a now sizable class of compounds invented by G. Lynch and G. Rogers (Lynch, 2004a) widely used in preclinical work. Ampakines are positive allosteric modulators of AMPA-type glutamate receptors, the mediators of fast EPSCs throughout the central nervous system, via a binding pocket located in the interface between each of the two dimers formed by the tetrameric subunits of the receptors (Jin et al., 2005). This interaction stabilizes the dimeric configuration and slows deactivation and desensitization of the receptors following binding of released glutamate. As expected from this, they significantly prolong open time of the receptor channel, resulting in enhancement of EPSPs both in slices and *in vivo* (Lynch, 2004a; Arai and Kessler, 2007). Ampakines readily cross the blood brain barrier following peripheral administration and rapidly increase central AMPA receptor mediated EPSPs (Staubli et al., 1994a,b).

Administered acutely, ampakines enhance LTP and memory encoding across different species and testing paradigms (Granger et al., 1993; Staubli et al., 1994a; Shors et al., 1995; Rogan et al., 1997; Porrino et al., 2005; Lynch and Gall, 2006; Hamlyn et al., 2009; Hampson et al., 2009; Simmons et al., 2009; Lynch et al., 2011). However, our work with models of intellectual disability has been guided by a second effect they produce that is directly related to the substrate map: the drugs up-regulate the production of BDNF (Lauterborn et al., 2000, 2003). This is not an unexpected result because work by Gall and co-workers (Isackson et al., 1991; Gall, 1992), and other groups (Castrén et al., 1992; Dragunow et al., 1993; Castren et al., 1998), demonstrated that increased neuronal firing and calcium influx (Shieh et al., 1998), as would be caused by enhanced excitatory transmission, triggers transcription of BDNF mRNA. Given that BDNF figures prominently in the substrate map (Figure 5), we asked if increasing brain levels of the factor with an ampakine rescues actin polymerization and LTP in the models.

The most extensive tests of the question were conducted using different models of Huntington Disease. Those experiments produced striking results: in the CAG140 model, four daily injections with an ampakine elevated hippocampal BDNF levels as evaluated 24 h after the last treatment (when the short half-life drug was long removed from circulation) and at the same time point restored both TBS-induced actin polymerization and LTP consolidation (Simmons et al., 2009). Moreover, long-term object recognition memory was restored to near normal values in the drug-treated mutants. It is worth noting here that 7 weeks of daily ampakine injections beginning in the third post-natal week blocked the onset of Huntington pathology, including striatal shrinkage, in the R6/2 model of early onset Huntington Disease and restored motor performance in a pole climbing test to levels not detectably different from wild type mice (Simmons et al., 2011).

The 4 day ampakine treatment regimen also increased BDNF levels in middle-aged rats and produced the same recovery of LTP found with acute administrations of the neurotrophin (Rex et al., 2006). Similarly, daily injections fully rescued TBS-induced actin polymerization, LTP, and context fear memory in the *Ube3a* mouse model of Angelman syndrome, again in tests carried out 24 h after the last injection of the short half-life compound (Baudry et al., 2012) (Figure 6). Finally, and perhaps most remarkably, ampakine pretreatment reinstated the TBS-induced RhoA-cofilin signaling, actin polymerization, and LTP consolidation that are otherwise severely impaired in middle-aged rats with long-term ovariectomy (Kramar et al., 2012a).

It remains for future studies on these rodent models to take full advantage of the opportunities afforded by the substrate map. In particular, and with the notable exception of chronically reduced estrogen, we have not established that the drug corrects specific signaling defects. In all of the studies, the ampakine did not affect baseline physiological properties but whether this holds for the multiple signaling cascades set in motion by TBS remains to be determined. Put simply, we cannot now say that the impressive rescue effects are due to rectification of specific impairments as opposed to activation of alternative pathways. Nor have we determined how broadly the ampakine affects transcription of actin regulatory proteins other than BDNF. Finally, potential ampakine effects on other forms of synaptic plasticity, such as long-term depression (LTD), are not understood. A single study evaluating this point found that one of two early variants potentiated NMDA receptor-dependent LTD induced by extended trains of paired heterosynaptic stimulation (Arai et al., 2004). With these caveats in mind, the results so far collected are encouraging with regard to the possibility of treatments that act across a broad spectrum of learning disorders.

The ampakine strategy for rescuing memory encoding focuses on the earliest post-synaptic step—fast, receptor mediated excitatory currents—in the complex sequences leading to reorganization of the subsynaptic cytoskeleton. An interesting, alternative approach would be to target downstream steps that are more directly related to actin management. This could potentially leave moment-to-moment synaptic communication unchanged while overriding defects in the machinery that produces lasting modifications to individual contacts. Two major difficulties facing such approaches involve (1) the lack of specificity of agents acting on enzymes or specific protein-protein interactions, and (2) the fact that downstream signaling events involved in LTP (e.g., activation of Src, ERK1/2) are ubiquitous and critical to many types of cell functions. Nonetheless, there are reasons to be optimistic that new technologies will eventually overcome these barriers.

Related to this, the ampakine strategy begins with the assumption that synaptic signaling defects associated with learning impairments are partial and embedded in a set of higher threshold, redundant pathways. In accord with this idea, enhanced synaptic drive during patterned afferent activity, as expected with ampakine treatment, might overcome an elevated threshold in a defective link in signaling, or engage a normally less responsive parallel cascade. Experiments in progress are evaluating these possibilities.

## Cognitive enhancement in normals

The many experiments showing improved memory with diverse manipulations in different species and across many types of learning, though not yet having translated into an approved human treatment, strongly suggest that synaptic encoding machinery is not maximally efficient under normal circumstances. With regard to mechanisms, many of the compounds found to enhance memory are also reported to facilitate the induction of LTP, most typically by indirectly facilitating NMDA receptor mediated currents. A critical but rarely discussed question concerns any possible costs of cognitive, or more specifically “encoding,” enhancement either at the neurobiological level or in behavioral terms. We take up of this point in the following section and then move to a consideration of the relationship of memory facilitation to cognitive enhancement. Finally, after having reviewed a sizable number of studies, we return to the issue (raised above) of why rodent studies on cognitive enhancement have such a poor record with regard to predicting human outcomes.

### LTP enhancement: robbing peter to pay paul?

**Figure 7:**
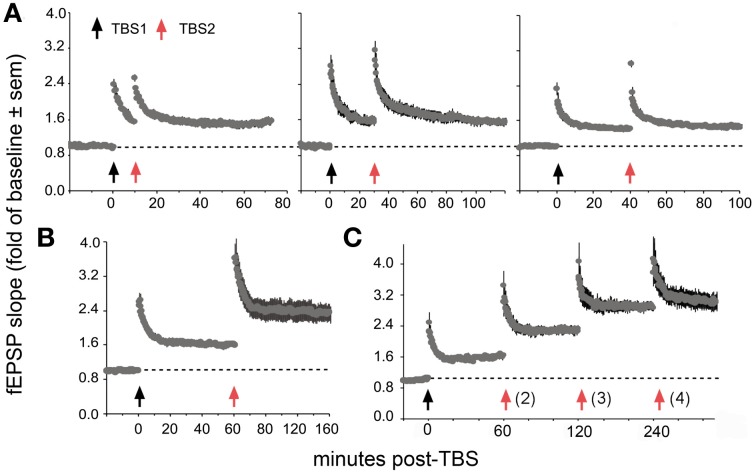
**Spaced stimulation augments LTP but only with delays of about 1 h.** Plots show effects of a first (TBS1) and second (TBS2) round of theta stimulation on fEPSP responses. **(A)** TBS1 (black arrow) reliably increases fEPSPs by about 50% above baseline whereas TBS2 (red arrow) applied 10 (left), 30 (middle) and 40 (right) min later does not augment the level of potentiation. **(B)** TBS2 applied 60 min after TBS1 doubles the level of potentiation. **(C)** TBS3 further augments potentiation if delayed by 60 min whereas a similarly delayed TBS4 has little effect suggesting that potentiation approaches ceiling levels after 3 spaced theta trains. Modified from Kramar et al. (2012b).

Recent work has uncovered surprising new TBS timing rules in field CA1 of rat hippocampal slices, the site and preparation most widely used in studies of LTP. Specifically, delivery of a second TBS train (TBS2) after a 50–60 min delay, *but not earlier*, doubles the magnitude of potentiation produced by TBS1 (Figure 7). TBS3, again delayed by 1 h further increases percent LTP while TBS4 is largely ineffective (Kramar et al., 2012b). TBS2 is also effective 90 min post-TBS1 and work from Frey and colleagues, using a more complex stimulation protocol, suggests that “LTP2” can be elicited with 4 h delays (Frey et al., 1995). These results describe a synaptic phenomenon that is roughly analogous to a fundamental property of learning; namely, that encoding is substantially improved by dividing training across a series of widely spaced trials (Wickelgren, 1974; Cepeda et al., 2006; Kornell and Bjork, 2008; Callan and Schweighofer, 2010). This spaced trials, or “distributed practice,” effect is strikingly evident for contextual fear conditioning in rats: learning is markedly improved by using training episodes separated by 1 h relative to that achieved with the same training presented in a massed session (Scharf et al., 2002).

**Figure 8:**
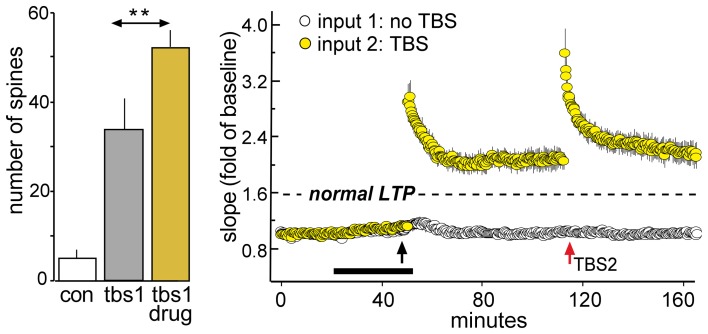
**Enhancing LTP1 blocks further increases in potentiation with a second round of TBS.** Theta burst stimulation (TBS) was applied to the Schaffer-commissural afferents to field CA1b in adult rat hippocampal slices. **Left**: Counts of densely phalloidin-labeled spines in the CA1 field of afferent stimulation show that in the presence of the ampakine CX614 (drug), one round of theta burst stimulation (tbs1) significantly increases the numbers of spines containing this marker of potentiation above that induced by TBS alone (^**^p < 0.01). **Right**: Plot of field EPSPs shows that TBS1 (black arrow) applied in the presence of ampakine infusion (black bar) elicits an LTP effect that is about twice as large as that produced in the absence of the drug (indicated by dashed line) but a second TBS bout (TBS2, red arrow) elicits no further potentiation (in contrast to effects of TBS2 applied under control conditions; see Figure 7B). Modified from Kramar et al. (2012b).

How do enhancing treatments interact with the LTP spaced trials effect? Acute administration of ampakines both lowers the threshold and in some cases raises the ceiling for LTP1 (Staubli et al., 1994a,b; Arai et al., 1996, 2004; Kramar et al., 2012b). We confirmed this effect by showing that in adult rat hippocampal slices a brief ampakine infusion during TBS1 greatly increased the percent potentiation (i.e., the magnitude of LTP1) but then found that LTP2, tested 1 h later and after ampakine washout, was absent (Figure 8) (Kramar et al., 2012b). In a sense, then, a single TBS train in the presence of the ampakine produced both LTP1 and LTP2, at the expense of further response enhancement by a second stimulation train. This result is interpretable in light of evidence that under normal circumstances TBS2 greatly expands the population of spines containing polymerized actin, indicating that it induces potentiation in synapses that failed to potentiate in response to TBS1. These observations further suggest that the sampled hippocampal field contains large populations of contacts with high plasticity thresholds. The argument has been confirmed in studies using the LTP-like effect produced by single spine glutamate uncaging. While direct stimulation produced structural changes to nearly all spines in immature hippocampal slices, in accord with earlier work (Matsuzaki et al., 2004; Harvey and Svoboda, 2007; Harvey et al., 2008), it affected less than half of the tested spines in adult slices (Kramar et al., 2012b).

A number of laboratories have described memory enhancement produced by acute ampakines in rodents, rabbits and primates, and as tested in a broad range of behavioral paradigms (Shors et al., 1995; Porrino et al., 2005; Arai and Kessler, 2007; Hampson et al., 2009; Lynch et al., 2011). But, with a few exceptions noted below, all of this work has used single training trials or daily sessions dealing with different problems. There appear to be no data relating to the effects of ampakines or other drugs in the context of behavioral practices used routinely, both in research and common practice, to improve memory. A collapsing of the benefits of distributed practice on encoding into a single trial, as expected from the LTP work, would greatly accelerate the acquisition of strong memory but would also eliminate any computational advantages of spacing. One psychological hypothesis for the origins of the ubiquitous spaced trials phenomenon posits that spacing restricts the amount of transient, noisy signals in the final memory. In all, it will be important in future work to evaluate costs and benefits of LTP-related memory enhancers in circumstances where timing rules are used to maximize the potentiation effect and long-term memory.

There may be ways of enhancing LTP without sacrificing the benefits of spaced training. Specifically, there is evidence that individual synapses have multiple levels of LTP (Enoki et al., 2009); if so, then it might be possible to increase potentiation in low threshold synapses without involving the high threshold contacts used to produce LTP2 and LTP3. This purely speculative proposition requires the assumption that some factor that “caps” the amount of potentiation is as work in a conventional, full length train of theta bursts. Removing this cap would then enhance LTP1 without eliminating LTP2. Given the results for ampakines, success in testing this string of arguments would likely require LTP enhancers that act independently of NMDA receptors. Estrogen represents a good example of a compound that satisfies this requirement because it facilitates elements in the substrate map (specifically, the RhoA-cofilin sequence) and LTP but not NMDA receptor currents (Kramar et al., 2009b).

### Memory drugs and cognitive enhancement

If we think of cognition as a process that organizes thoughts and future actions by integrating new information with already learned material, then there is no evident reason to assume that it will be enhanced by manipulations that strengthen memory encoding. But this perspective ignores the strong likelihood that the networks underlying cognitive activities are themselves products of learning. Thus, two questions about enhancers and cognition emerge: (1) Do repeated administrations of memory promoters across many learning sessions result in new intellectual capabilities? (2) Does acute administration with such agents produce evidence of enhanced cognition? There are data relating to the first point. Hampson, Deadwyler and colleagues (Hampson et al., 1998) trained rats for weeks until asymptotic performance was reached on a complex non-match to sample problem and then introduced every other day injections of an ampakine. Gradually over 2 weeks the rats went well-beyond the asymptotic learning scores found in controls. Detailed analyses showed that this supra-normal performance reflected the acquisition of an ability not found in controls; specifically, a capacity to suppress the effects of mistakes on future learning. These results encourage the idea that memory enhancement can be used to form behaviorally useful cognitive structures that are beyond the range of control animals.

There are also reasons to think that memory enhancing drugs, or at least that category acting on fast EPSCs, will acutely increase cognitive capacities. It is generally assumed that cognition involves moment by moment assembly of cortical neurons into networks, from which it could be postulated that expanding those networks would lead to new capacities. Experimental work has established that ampakines, as expected from their mode of action, greatly facilitate throughput in the tri-synaptic intra-hippocampal circuit (Sirvio et al., 1996), in essence expanding the network engaged by activation of a single input. More impressively, it is reported from PET studies of well-trained monkeys that injection of an ampakine during a learning session results in spatially discrete activation of cortical regions that normally remain silent during a complex learning problem (Porrino et al., 2005). A fascinating aspect of these results is that the incorporated associational region (precuneus area of the midline cortex) is thought from human work to be a site critical to mental imagining of the response about to be performed. Notably, learning scores were greatly improved in drug vs. vehicle trials in the same monkey.

It will be recognized that the above results involved single studies and that obvious follow up work is lacking. They do, however, accord with results showing that pharmacologically increasing fast, excitatory transmission expands networks in slices; it is thus reasonable to consider the results as significant evidence for the possibility of elaborating the momentary cortical structures used to address complex problems.

It is widely held that extended training serves to reduce the number of neurons required to process a given type of complex information (e.g., Hess et al., 2003; Karlsson and Frank, 2008). Thus, learning not only encodes information but increases the efficiency of computation as well. Why, then, would a drug that elevates activity within a field, and possibly incorporates normally inactive regions into a cortical pattern, enhance performance in a learning trial? The recording and imaging studies of ampakine effects on learning have, to date, used well-trained animals dealing with complex problems of a very familiar type; we can assume that these subjects have already minimized the cortical expenditures needed for good learning scores. We argue that breaking through the normal ceiling on performance under these conditions can be achieved by adding neuronal resources under the guidance of highly efficient (minimized), stable networks built up over many training sessions. The hypothesis is therefore one in which learning is posited to create, via LTP-like mechanisms, circuits that are gradually stripped of extraneous elements by further training, while enhancement beyond normal limits involves transiently and selectively extending these dominant circuits.

The neural network theory arguments for the above idea are familiar: the output from a select population of intensely active cells converges on a second group of neurons but is not sufficient to bring the targets to firing threshold. Ampakines, by increasing the potency of transmission, allow the learned responses of the initial cells to activate the secondary group. The expanded system, driven by a core of sharpened networks, could add new capabilities, rather than noise, to cortical processing of complex inputs.

### Missing features in animal studies of memory and cognition enhancement

Translation is a primary goal of preclinical work on enhancement and it is appropriate to consider a few of what are likely to be many conditions involved in testing human cognition that are missing from animal studies. First, and as mentioned earlier, the animal experiments are concerned with problem solving in part because of the need for easily quantified behaviors and, perhaps, the history of experimental psychology. It is not at all clear that the solving of particular problems, even ones that are difficult, represents the bulk of human cognition. Thought involves the integration of a vast amount of material, shuffling of the resultant assemblies, and then another attempt at a satisfactory integration. Salient cues, a limited number of defined choices, and predetermined rewards are largely lacking in this activity, appropriately described by the 18th century philosopher Kant as “free play of the cognitive powers” (Kant, 1987). It is not obvious, at least to us, that enhancing scores on puzzles or tasks involving particular computations will be accompanied by improvements in the fluid world of the imagination.

Second, the inner environment of humans is one of enormous complexity, with vast amounts of readily accessible memories, resulting in a correspondingly large number of free choices at each step of cognition. We can safely assume that nothing like this is present in rodents, a point that encapsulates the great difficulty of predicting human outcomes from preclinical results obtained with putative cognitive enhancers. Still, rats are proficient in dealing with a great amount of *external* complexity as contained, for example, in real world environments. It is somewhat surprising then that proposed enhancers aren't pre-clinically screened in animals exposed to circumstances where the number of free choices is high and behavior is unsupervised, somewhat in the manner that holds for human thought. Complex environments would allow for analysis of how behavior is self-organized into sequences and whether a potential cognitive enhancer either accelerates this process or affects the length and branching of such movement series. A practical advantage would be that the many different behaviors exhibited by rats in these conditions would allow for robust analysis of drug selectivity and side effects.

Third, humans bring a great deal of past experience with dense internal and external worlds to bear on in-the-moment cognitive activity. Yet, rats used in enhancement studies, though usually extensively handled, have nothing like this advantage of experience when introduced to a testing apparatus. While it is true that many testing paradigms involve multiple sessions or, in a few cases, extensive pre-training, there is evidence that a backlog of generalized experience with rich environments can profoundly affect subsequent performance in novel circumstances. This could reflect the multiply reported effects of prolonged stays in enriched environments on forebrain microanatomy and neurochemistry; put simply, environmental enrichment might be needed to finish brain development and create something like a normal state. If so, then the great majority of cognitive enhancement studies work with a sub-optimal neurobiological preparation, something that would reduce their relevance to the fully differentiated human brain. Alternatively, extended interactions with environmental complexity, acting through LTP-like mechanisms, could result in the creation of cortical networks and stable cognitive paradigms that are applicable to virtually any type of situation. Laboratory rats lacking these structures normally arising from many encounters with real world environments would constitute a badly flawed model regarding applicability to humans.

### Conflict of interest statement

Gary Lynch and Christine M. Gall are listed as inventors on an awarded patent, held by the University of California, for the use ampakines to upregulate BDNF expression and for facilitation of learning.

## References

[B1] Abraham W. C. (2003). How long will long-term potentiation last. Philos. Trans. R. Soc. Lond. B Biol. Sci. 358, 735–744 10.1098/rstb.2002.1222 12740120 PMC1693170

[B2] Ackerley S. J. Stinear C. M. Barber P. A. Byblow W. D. (2010). Combining theta burst stimulation with training after subcortical stroke. Stroke 41, 1568–1572 10.1161/STROKEAHA.110.583278 20489170

[B3] Arai A. Kessler M. Rogers G. Lynch G. (1996). Effects of a memory enhancing drug on AMPA receptor currents and synaptic transmission in hippocampus. J. Pharmacol. Exp. Ther. 278, 627–638 8768713

[B4] Arai A. C. Kessler M. (2007). Pharmacology of ampakine modulators: from AMPA receptors to synapses and behavior. Curr. Drug Targets 8, 583–602 10.2174/138945007780618490 17504103

[B5] Arai A. C. Xia Y. F. Suzuki E. (2004). Modulation of AMPA receptor kinetics differentially influences synaptic plasticity in the hippocampus. Neuroscience. 123, 1011–1024 10.1016/j.neuroscience.2003.10.033 14751292

[B6] Axmacher N. Mormann F. Fernandez G. Elger C. E. Fell J. (2006). Memory formation by neuronal synchronization. Brain Res. Rev. 52, 170–182 10.1016/j.brainresrev.2006.01.007 16545463

[B7] Babayan A. H. Kramar E. A. Barrett R. M. Jafari M. Haettig J. Chen L. Y. (2012). Integrin dynamics produce a delayed stage of long-term potentiation and memory consolidation. J. Neurosci. 32, 12854–12861 10.1523/JNEUROSCI.2024-12.2012 22973009 PMC3752079

[B8] Baudry M. Kramar E. Xu X. Zadran H. Moreno S. Lynch G. (2012). Ampakines promote spine actin polymerization, long-term potentiation, and learning in a mouse model of angelman syndrome. Neurobiol. Dis. 47, 210–215 10.1016/j.nbd.2012.04.002 22525571 PMC3367059

[B9] Belichenko P. V. Kleschevnikov A. M. Salehi A. Epstein C. J. Mobley W. C. (2007). Synaptic and cognitive abnormalities in mouse models of down syndrome: exploring genotype-phenotype relationships. J. Comp. Neurol. 504, 329–345 10.1002/cne.21433 17663443

[B10] Bliss T. V. Collingridge G. L. (1993). A synaptic model of memory: long-term potentiation in the hippocampus. Nature 361, 31–39 10.1038/361031a0 8421494

[B11] Bozdagi O. Nagy V. Kwei K. T. Huntley G. W. (2007). *In vivo* roles for matrix metalloproteinase-9 in mature hippocampal synaptic physiology and plasticity. J. Neurophysiol. 98, 334–344 10.1152/jn.00202.2007 17493927 PMC4415272

[B12] Brakebusch C. Fassler R. (2003). The integrin-actin connection, an eternal love affair. Embo. J. 22, 2324–2333 10.1093/emboj/cdg245 12743027 PMC156003

[B13] Buzsaki G. (2005). Theta rhythm of navigation: link between path integration and landmark navigation, episodic and semantic memory. Hippocampus 15, 827–840 10.1002/hipo.20113 16149082

[B14] Cakic V. (2009). Smart drugs for cognitive enhancement: ethical and pragmatic considerations in the era of cosmetic neurology. J. Med. Ethics. 35, 611–615 10.1136/jme.2009.030882 19793941

[B15] Callan D. E. Schweighofer N. (2010). Neural correlates of the spacing effect in explicit verbal semantic encoding support the deficient-processing theory. Hum. Brain Mapp. 31, 645–659 19882649 10.1002/hbm.20894 PMC6871263

[B16] Cao C. Rioult-Pedotti M. S. Migani P. Yu C. J. Tiwari R. Parang K. (2013). Impairment of TrkB-PSD-95 signaling in angelman syndrome. PLoS Biol. 11:e1001478 10.1371/journal.pbio.1001478 23424281 PMC3570550

[B17] Castren E. Berninger B. Leingartner A. Lindholm D. (1998). Regulation of brain derived neurotrophic factor mRNA levels in hippocampus by neuronal activity. Prog. Brain Res. 117, 57–64 10.1016/S0079-6123(08)64007-8 9932400

[B18] Castrén E. Zafra F. Thoenen H. Lindholm D. (1992). Light regulates expression of brain-derived neurotrophic factor mRNA in rat visual cortex. Proc. Natl. Acad. Sci. U.S.A. 89, 9444–9448 10.1073/pnas.89.20.9444 1409655 PMC50148

[B19] Cepeda N. J. Pashler H. Vul E. Wixted J. T. Rohrer D. (2006). Distributed practice in verbal recall tasks: a review and quantitative synthesis. Psychol. Bull. 132, 354–380 10.1037/0033-2909.132.3.354 16719566

[B20] Chan C. S. Levenson J. M. Mukhopadhyay P. S. Zong L. Bradley A. Sweatt J. D. (2007). Alpha3-integrins are required for hippocampal long-term potentiation and working memory. Learn. Mem. 14, 606–615 10.1101/lm.648607 17848500 PMC1994082

[B21] Chan C.-S. Weeber E. J. Kurup S. Sweatt J. D. Davis R. L. (2003). Integrin requirement for hippocampal synaptic plasticity and spatial memory. J. Neurosci. 23, 7107–7116 12904471 10.1523/JNEUROSCI.23-18-07107.2003 PMC6740650

[B22] Chan C. S. Weeber E. J. Zong L. Fuchs E. Sweatt J. D. Davis R. L. (2006). Beta 1-integrins are required for hippocampal AMPA receptor-dependent synaptic transmission, synaptic plasticity, and working memory. J. Neurosci. 26, 223–232 10.1523/JNEUROSCI.4110-05.2006 16399691 PMC2408376

[B23] Chen L. Y. Rex C. S. Babayan A. H. Kramar E. A. Lynch G. Gall C. M. (2010). Physiological activation of synaptic Rac>PAK (p-21 activated kinase) signaling is defective in a mouse model of fragile x syndrome. J. Neurosci. 30, 10977–10984 10.1523/JNEUROSCI.1077-10.2010 20720104 PMC2929244

[B24] Chen L. Y. Rex C. S. Casale M. S. Gall C. M. Lynch G. (2007). Changes in synaptic morphology accompany actin signaling during LTP. J. Neurosci. 27, 5363–5372 10.1523/JNEUROSCI.0164-07.2007 17507558 PMC6672340

[B25] Chen Y. Kramar E. A. Chen L. Y. Babayan A. H. Andres A. L. Gall C. M. (2013). Impairment of synaptic plasticity by the stress mediator CRH involves selective destruction of thin dendritic spines via RhoA signaling. Mol. Psychiatry 18, 485–496 10.1038/mp.2012.17 22411227 PMC3440527

[B26] Chun D. Gall C. M. Bi X. Lynch G. (2001). Evidence that integrins contribute to multiple stages in the consolidation of long term potentiation. Neuroscience 105, 815–829 10.1016/S0306-4522(01)00173-7 11530220

[B27] Costa A. C. Grybko M. J. (2005). Deficits in hippocampal CA1 LTP induced by TBS but not HFS in the Ts65Dn mouse: A model of down syndrome. Neurosci. Lett. 382, 317–322 10.1016/j.neulet.2005.03.031 15925111

[B28] Danen E. H. van Rheenen J. Franken W. Huveneers S. Sonneveld P. Jalink K. (2005). Integrins control motile strategy through a Rho-cofilin pathway. J. Cell Biol. 169, 515–526 10.1083/jcb.200412081 15866889 PMC2171933

[B29] Darby R. (2010). Ethical issues in the use of cognitive enhancement. Pharos. Alpha Omega Alpha Honor. Med. Soc. 73, 16–22 20455376

[B30] Dash P. K. Orsi S. A. Moody M. Moore A. N. (2004). A role for hippocampal Rho-ROCK pathway in long-term spatial memory. Biochem. Biophys. Res. Commun. 322, 893–898 10.1016/j.bbrc.2004.08.004 15336547

[B31] Davis M. Hanson S. A. Altevogt B. (2008). Neuroscience Biomarkers and Biosignatures. Converging Technologies, Emerging Partnerships: Workshop Summary. Washington, DC: National Academies Press 21452450

[B32] del Toro D. Canals J. M. Gines S. Kojima M. Egea G. Alberch J. (2006). Mutant huntingtin impairs the post-golgi trafficking of brain-derived neurotrophic factor but not its val66met polymorphism. J. Neurosci. 26, 12748–12757 10.1523/JNEUROSCI.3873-06.2006 17151278 PMC6674849

[B33] DeMali K. A. Wennerberg K. Burridge K. (2003). Integrin signaling to the actin cytoskeleton. Curr. Opin. Cell Biol. 15, 572–582 10.1016/S0955-0674(03)00109-1 14519392

[B34] Demeter E. Sarter M. Lustig C. (2008). Rats and humans paying attention: cross-species task development for translational research. Neuropsychology 22, 787–799 10.1037/a0013712 18999353 PMC2705465

[B35] Dragunow M. Beilharz E. Mason B. Lawlor P. Abraham W. Gluckman P. (1993). Brain-derived neurotrophic factor expression after long-term potentiation. Neurosci. Lett. 160, 232–236 10.1016/0304-3940(93)90420-P 8247360

[B36] Ehninger D. Han S. Shilyansky C. Zhou Y. Li W. Kwiatkowski D. J. (2008). Reversal of learning deficits in a Tsc2+/− mouse model of tuberous sclerosis. Nat. Med. 14, 843–848 10.1038/nm1788 18568033 PMC2664098

[B37] Enoki R. Hu Y. L. Hamilton D. Fine A. (2009). Expression of long-term plasticity at individual synapses in hippocampus is graded, bidirectional, and mainly presynaptic: optical quantal analysis. Neuron 62, 242–253 10.1016/j.neuron.2009.02.026 19409269

[B38] Farah M. J. Haimm C. Sankoorikal G. Smith M. E. Chatterjee A. (2009). When we enhance cognition with Adderall, do we sacrifice creativity. A preliminary study. Psychopharmacology (Berl.) 202, 541–547 10.1007/s00213-008-1369-3 19011838

[B39] Forlini C. Hall W. Maxwell B. Outram S. M. Reiner P. B. Repantis D. (2013). Navigating the enhancement landscape. Ethical issues in research on cognitive enhancers for healthy individuals. EMBO Rep. 14, 123–128 10.1038/embor.2012.225 23318628 PMC3566849

[B40] Frey U. Schollmeier K. Reymann K. G. Seidenbecher T. (1995). Asymptotic hippocampal long-term potentiation in rats does not preclude additional potentiation at later phases. Neuroscience 67, 799–807 10.1016/0306-4522(95)00117-2 7675206

[B41] Fulton J. (1941). Encephalization of motor functions during the evolution of the primate nervous system. Ohio J. Sci. 41, 173–182 8657555

[B42] Gall C. (1992). Regulation of brain neurotrophin expression by physiological activity. Trends Pharmacol. Sci. 13, 401–403 10.1016/0165-6147(92)90123-N 1440876

[B43] Gavin C. F. Rubio M. D. Young E. Miller C. Rumbaugh G. (2012). Myosin II motor activity in the lateral amygdala is required for fear memory consolidation. Learn. Mem. 19, 9–14 10.1101/lm.024042.111 22174310 PMC3246591

[B44] Granger R. Staubli U. Davis M. Perez Y. Nillson L. Rogers G. A. (1993). A drug that facilitates glutamatergic transmission reduces exploratory activity and improves performance in a learning-dependent task. Synapse 15, 326–329 10.1002/syn.890150409 8153879

[B45] Hamlyn E. Brand L. Shahid M. Harvey B. H. (2009). The ampakine, Org 26576, bolsters early spatial reference learning and retrieval in the morris water maze: a subchronic, dose-ranging study in rats. Behav. Pharmacol. 20, 662–667 10.1097/FBP.0b013e328331ba1b 19741506

[B46] Hampson R. E. Espana R. A. Rogers G. A. Porrino L. J. Deadwyler S. A. (2009). Mechanisms underlying cognitive enhancement and reversal of cognitive deficits in nonhuman primates by the ampakine CX717. Psychopharmacology (Berl.) 202, 355–369 10.1007/s00213-008-1360-z 18985324 PMC3107999

[B47] Hampson R. E. Rogers G. Lynch G. Deadwyler S. A. (1998). Facilitative effects of the ampakine CX516 on short-term memory in rats: enhancement of delayed-nonmatch-to-sample performance. J. Neurosci. 18, 2740–2747 9502831 10.1523/JNEUROSCI.18-07-02740.1998 PMC6793095

[B48] Harvey C. D. Svoboda K. (2007). Locally dynamic synaptic learning rules in pyramidal neuron dendrites. Nature. 450, 1195–1200 10.1038/nature06416 18097401 PMC3425382

[B49] Harvey C. D. Yasuda R. Zhong H. Svoboda K. (2008). The spread of Ras activity triggered by activation of a single dendritic spine. Science. 321, 136–140 10.1126/science.1159675 18556515 PMC2745709

[B50] Hess U. Whalen S. Sandoval L. Lynch G. Gall C. (2003). Ampakines reduce methamphetamine-driven rotation and activate neocortex in a regionally selective fashion. Neuroscience 121, 509–521 10.1016/S0306-4522(03)00423-8 14522010

[B51] Hsu Y. F. Liao K. K. Lee P. L. Tsai Y. A. Yeh C. L. Lai K. L. (2011). Intermittent theta burst stimulation over primary motor cortex enhances movement-related beta synchronisation. Clin. Neurophysiol. 122, 2260–2267 10.1016/j.clinph.2011.03.027 21543254

[B52] Huang C. C. Liang Y. C. Hsu K. S. (1999). A role for extracellular adenosine in time-dependent reversal of long-term potentiation by low-frequency stimulation at hippocampal CA1 synapses. J. Neurosci. 19, 9728–9738 10559382 10.1523/JNEUROSCI.19-22-09728.1999 PMC6782980

[B53] Huang Y. Z. Rothwell J. C. Chen R. S. Lu C. S. Chuang W. L. (2011). The theoretical model of theta burst form of repetitive transcranial magnetic stimulation. Clin. Neurophysiol. 122, 1011–1018 10.1016/j.clinph.2010.08.016 20869307 PMC3046904

[B54] Huang Z. Shimazu K. Woo N. H. Zang K. Muller U. Lu B. (2006). Distinct roles of the beta 1-class integrins at the developing and the mature hippocampal excitatory synapse. J. Neurosci. 26, 11208–11219 10.1523/JNEUROSCI.3526-06.2006 17065460 PMC2693048

[B55] Hyman S. E. (2011). Cognitive enhancement: promises and perils. Neuron 69, 595–598 10.1016/j.neuron.2011.02.012 21338872

[B56] Isackson P. J. Murray K. Huntsman M. Gall C. M. (1991). BDNF mRNA expression is increased in adult rat forebrain after limbic seizures: temporal patterns of induction distinct from NGF. Neuron 6, 937–948 10.1016/0896-6273(91)90234-Q 2054188

[B57] Jiang Y. H. Armstrong D. Albrecht U. Atkins C. M. Noebels J. L. Eichele G. (1998). Mutation of the Angelman ubiquitin ligase in mice causes increased cytoplasmic p53 and deficits of contextual learning and long-term potentiation. Neuron 21, 799–811 10.1016/S0896-6273(00)80596-6 9808466

[B58] Jin R. Clark S. Weeks A. M. Dudman J. T. Gouaux E. Partin K. M. (2005). Mechanism of positive allosteric modulators acting on AMPA receptors. J. Neurosci. 25, 9027–9036 10.1523/JNEUROSCI.2567-05.2005 16192394 PMC6725607

[B59] Kant I. (1987) Critique of Judgement, Translated by Werner S. Pluhar. Indianapolis: Hackett Publishing Co

[B60] Karlsson M. P. Frank L. M. (2008). Network dynamics underlying the formation of sparse, informative, representations in the hippocampus. J. Neurosci. 28, 14271–14281 10.1523/JNEUROSCI.4261-08.2008 19109508 PMC2632980

[B61] Kornell N. Bjork R. A. (2008). Learning concepts and categories: is spacing the “Enemy of induction.” Psychol. Sci. 19, 585–592 10.1111/j.1467-9280.2008.02127.x 18578849

[B62] Kovalchuk Y. Hanse E. Kafitz K. W. Konnerth A. (2002). Postsynaptic induction of BDNF-mediated long-term potentiation. Science 295, 1729–1735 10.1126/science.1067766 11872844

[B63] Kramar E. A. Babayan A. H. Gall C. M. Lynch G. (2013). Estrogen promotes learning-related plasticity by modifying the synaptic cytoskeleton. Neuroscience 239, 3–16 10.1016/j.neuroscience.2012.10.038 23103216 PMC4472431

[B64] Kramar E. A. Chen L. Y. Lauterborn J. C. Simmons D. A. Gall C. M. Lynch G. (2012a). BDNF upregulation rescues synaptic plasticity in middle-aged ovariectomized rats. Neurobiol. Aging 33, 708–719 10.1016/j.neurobiolaging.2010.06.008 20674095 PMC2978788

[B65] Kramar E. A. Babayan A. H. Gavin C. F. Cox C. D. Jafari M. Gall C. M. (2012b). Synaptic evidence for the efficacy of spaced learning. Proc. Natl. Acad. Sci. U.S.A. 109, 5121–5126 10.1073/pnas.1120700109 22411798 PMC3323981

[B66] Kramar E. A. Bernard J. A. Gall C. M. Lynch G. (2002). Alpha3 integrin receptors contribute to the consolidation of long-term potentiation. Neuroscience 110, 29–39 10.1016/S0306-4522(01)00540-1 11882370

[B67] Kramar E. A. Chen L. Y. Rex C. S. Gall C. M. Lynch G. (2009a). Estrogen's place in the family of synaptic modulators. Mol. Cell Pharmacol. 1, 258–262 20419049 PMC2858427

[B68] Kramar E. A. Chen L. Y. Brandon N. J. Rex C. S. Liu F. Gall C. M. (2009b). Cytoskeletal changes underlie estrogen's acute effects on synaptic transmission and plasticity. J. Neurosci. 29, 12982–12993 10.1523/JNEUROSCI.3059-09.2009 19828812 PMC2806054

[B69] Kramar E. A. Lin B. Rex C. S. Gall C. M. Lynch G. (2006). Integrin-driven actin polymerization consolidates long-term potentiation. Proc. Natl. Acad. Sci. U.S.A. 103, 5579–5584 10.1073/pnas.0601354103 16567651 PMC1459396

[B70] Krucker T. Siggins G. R. Halpain S. (2000). Dynamic actin filaments are required for stable long-term potentiation (LTP) in area CA1 of the hippocampus. Proc. Natl. Acad. Sci. U.S.A. 97, 6856–6861 10.1073/pnas.100139797 10823894 PMC18765

[B71] Lamprecht R. (2011). The roles of the actin cytoskeleton in fear memory formation. Front. Behav. Neurosci. 5:39 10.3389/fnbeh.2011.00039 21808614 PMC3139223

[B72] Larson J. Lynch G. (1986). Induction of synaptic potentiation in hippocampus by patterned stimulation involves two events. Science 232, 985–988 10.1126/science.3704635 3704635

[B73] Larson J. Wong D. Lynch G. (1986). Patterned stimulation at the theta frequency is optimal for the induction of hippocampal long-term potentiation. Brain Res. 368, 347–350 10.1016/0006-8993(86)90579-2 3697730

[B74] Larson J. Xiao P. Lynch G. (1993). Reversal of LTP by theta frequency stimulation. Brain Res. 600, 97–102 10.1016/0006-8993(93)90406-D 8422592

[B75] Lauterborn J. Troung G. Baudry M. Bi X. Lynch G. Gall C. (2003). Chronic elevation of brain-derived neurotrophic factor by ampakines. J. Pharmacol. Exp. Ther. 307, 297–305 10.1124/jpet.103.053694 12893840

[B76] Lauterborn J. C. Lynch G. Vanderklish P. Arai A. Gall C. M. (2000). Positive modulation of AMPA receptors increases neurotrophin expression by hippocampal and cortical neurons. J. Neurosci. 20, 8–21 10627576 10.1523/JNEUROSCI.20-01-00008.2000 PMC6774091

[B77] Lauterborn J. C. Rex C. S. Kramar E. Chen L. Y. Pandyarajan V. Lynch G. (2007). Brain-derived neurotrophic factor rescues synaptic plasticity in a mouse model of fragile X syndrome. J. Neurosci. 27, 10685–10694 10.1523/JNEUROSCI.2624-07.2007 17913902 PMC6672822

[B78] Lee H. Y. Ge W. P. Huang W. He Y. Wang G. X. Rowson-Baldwin A. (2011). Bidirectional regulation of dendritic voltage-gated potassium channels by the fragile X mental retardation protein. Neuron 72, 630–642 10.1016/j.neuron.2011.09.033 22099464 PMC3433402

[B79] Lin B. Kramar E. A. Bi X. Brucher F. A. Gall C. M. Lynch G. (2005). Theta stimulation polymerizes actin in dendritic spines of hippocampus. J. Neurosci. 25, 2062–2069 10.1523/JNEUROSCI.4283-04.2005 15728846 PMC6726058

[B80] Lynch G. (2004a). AMPA receptor modulators as cognitive enhancers. Curr. Opin. Pharmacol. 4, 4–11 10.1016/j.coph.2003.09.009 15018832

[B81] Lynch M. A. (2004b). Long-term potentiation and memory. Physiol. Rev. 84, 87–136 10.1152/physrev.00014.2003 14715912

[B82] Lynch G. Baudry M. (1988). The biochemistry of memory: a new and specific hypothesis. Science 224, 1057–1063 10.1126/science.6144182 6144182

[B83] Lynch G. Gall C. M. (2006). Ampakines and the threefold path to cognitive enhancement. Trends Neurosci. 29, 554–562 10.1016/j.tins.2006.07.007 16890999

[B84] Lynch G. Granger R. (2008). Big Brain: The Origins and Future of Human Intelligence. New York, NY: Palgrave Macmillan Press

[B85] Lynch G. Kramar E. A. Rex C. S. Jia Y. Chappas D. Gall C. M. (2007). Brain-derived neurotrophic factor restores synaptic plasticity in a knock-in mouse model of Huntington's disease. J. Neurosci. 27, 4424–4434 10.1523/JNEUROSCI.5113-06.2007 17442827 PMC6672319

[B86] Lynch G. Palmer L. C. Gall C. M. (2011). The likelihood of cognitive enhancement. Pharmacol. Biochem. Behav. 99, 116–129 10.1016/j.pbb.2010.12.024 21215768 PMC3114293

[B87] Lynch G. Rex C. S. Chen L. Y. Gall C. M. (2008). The substrates of memory: defects, treatments, and enhancement. Eur. J. Pharmacol. 585, 2–13 10.1016/j.ejphar.2007.11.082 18374328 PMC2427007

[B88] Lynch G. Rex C. S. Chen L. Y. Gall C. M. (2010). Synaptic mechanisms for encoding memory, in Encyclopedia of Behavioral Neuroscience, Vol. 3, eds Koob G. Thompson R. F. Le Moal M. (Section ed) Shors T. (London: Academic Press, an imprint of Elsevier Ltd.), 356–364

[B89] Matsuzaki M. Honkura N. Ellis-Davies G. C. Kasai H. (2004). Structural basis of long-term potentiation in single dendritic spines. Nature 429, 761–766 10.1038/nature02617 15190253 PMC4158816

[B90] Menache A. (2012). Are animal models relevant in modern psychiatry. Psychiatr. Times 29, 1–5

[B91] Miranti C. K. Brugge J. S. (2002). Sensing the environment: a historical perspective on integrin signal transduction. Nat. Cell Biol. 4, E83–E90 10.1038/ncb0402-e83 11944041

[B92] Moretti P. Levenson J. M. Battaglia F. Atkinson R. Teague R. Antalffy B. (2006). Learning and memory and synaptic plasticity are impaired in a mouse model of rett syndrome. J. Neurosci. 26, 319–327 10.1523/JNEUROSCI.2623-05.2006 16399702 PMC6674314

[B93] Morris R. G. (2003). Long-term potentiation and memory. Philos.Trans. R. Soc. Lond. B Biol. Sci. 358, 643–647 10.1098/rstb.2002.1230 12740109 PMC1693171

[B94] Murray C. A. Lynch M. A. (1998). Evidence that increased hippocampal expression of the cytokine interleukin1ß is a common trigger for age- and stress-induced impairments in long-term potentiation. J. Neurosci. 18, 2974–2981 9526014 10.1523/JNEUROSCI.18-08-02974.1998 PMC6792583

[B94a] Murphy K. P. Carter R. J. Lione L. A. Mangiarini L. Mahal A. Bates G. P. (2000). Abnormal synaptic plasticity and impaired spatial cognition in mice transgenic for exon 1 of the human huntington's disease mutation. J. Neurosci. 20, 5115–5123 10864968 10.1523/JNEUROSCI.20-13-05115.2000 PMC6772265

[B95] Nagy V. Bozdagi O. Huntley G. W. (2007). The extracellular protease matrix metalloproteinase-9 is activated by inhibitory avoidance learning and required for long-term memory. Learn. Mem. 14, 655–664 10.1101/lm.678307 17909100 PMC2044557

[B96] Nagy V. Bozdagi O. Matynia A. Balcerzyk M. Okulski P. Dzwonek J. (2006). Matrix metalloproteinase-9 is required for hippocampal late-phase long-term potentiation and memory. J. Neurosci. 26, 1923–1934 10.1523/JNEUROSCI.4359-05.2006 16481424 PMC4428329

[B97] Otto T. Eichenbaum H. Wiener S. I. Wible C. G. (1991). Learning-related patterns of CA1 spike trains parallel stimulation parameters optimal for inducing hippocampal long-term potentiation. Hippocampus 1, 181–192 10.1002/hipo.450010206 1669292

[B98] Porrino L. Daunais J. Rogers G. Hampson R. Deadwyler S. (2005). Facilitation of task performance and removal of the effects of sleep deprivation by an ampakine (CX717) in nonhuman primates. PLoS Biol. 3:e299 10.1371/journal.pbio.0030299 16104830 PMC1188239

[B99] Rex C. S. Chen L. Y. Sharma A. Liu J. Babayan A. H. Gall C. M. (2009). Different Rho GTPase-dependent signaling pathways initiate sequential steps in the consolidation of long-term potentiation. J. Cell Biol. 186, 85–97 10.1083/jcb.200901084 19596849 PMC2712993

[B100] Rex C. S. Gavin C. F. Rubio M. D. Kramar E. A. Chen L. Y. Jia Y. (2010). Myosin IIb regulates actin dynamics during synaptic plasticity and memory formation. Neuron 67, 603–617 10.1016/j.neuron.2010.07.016 20797537 PMC2929390

[B101] Rex C. S. Kramar E. A. Colgin L. L. Lin B. Gall C. M. Lynch G. (2005). Long-term potentiation is impaired in middle-aged rats: regional specificity and reversal by adenosine receptor antagonists. J. Neurosci. 25, 5956–5966 10.1523/JNEUROSCI.0880-05.2005 15976084 PMC6724797

[B102] Rex C. S. Lauterborn J. C. Lin C. Y. Kramar E. A. Rogers G. A. Gall C. M. (2006). Restoration of long-term potentiation in middle-aged hippocampus after induction of brain-derived neurotrophic factor. J. Neurophysiol. 96, 677–685 10.1152/jn.00336.2006 16707719 PMC1554892

[B103] Rex C. S. Lin C. Y. Kramar E. A. Chen L. Y. Gall C. M. Lynch G. (2007). Brain-derived neurotrophic factor promotes long-term potentiation-related cytoskeletal changes in adult hippocampus. J. Neurosci. 27, 3017–3029 10.1523/JNEUROSCI.4037-06.2007 17360925 PMC6672589

[B104] Rogan M. T. Staubli U. V. LeDoux J. E. (1997). AMPA receptor facilitation accelerates fear learning without altering the level of conditioned fear acquired. J. Neurosci. 17, 5928–5935 9221789 10.1523/JNEUROSCI.17-15-05928.1997 PMC6573187

[B105] Sacktor T. C. (2008). PKMzeta, LTP maintenance, and the dynamic molecular biology of memory storage. Prog. Brain Res. 169, 27–40 10.1016/S0079-612300002-7 18394466

[B106] Sahakian B. J. Morein-Zamir S. (2011). Neuroethical issues in cognitive enhancement. J. Psychopharmacol. 25, 197–204 10.1177/0269881109106926 20212064

[B107] Sarter M. Martinez V. Kozak R. (2009). A neurocognitive animal model dissociating between acute illness and remission periods of schizophrenia. Psychopharmacology (Berl.) 202, 237–258 10.1007/s00213-008-1216-6 18618100 PMC2719245

[B108] Sauvage M. M. (2010). Roc in animals: uncovering the neural substrates of recollection and familiarity in episodic recognition memory. Conscious. Cogn. 19, 816–828 10.1016/j.concog.2010.06.023 20691613 PMC2956179

[B109] Sauvage M. M. Fortin N. J. Owens C. B. Yonelinas A. P. Eichenbaum H. (2008). Recognition memory: opposite effects of hippocampal damage on recollection and familiarity. Nat. Neurosci. 11, 16–18 10.1038/nn2016 18037884 PMC4053160

[B110] Scharf M. T. Woo N. H. Lattal K. M. Young J. Z. Nguyen P. V. Abel T. (2002). Protein synthesis is required for the enhancement of long-term potentiation and long-term memory by spaced training. J. Neurophysiol. 87, 2770–2777 12037179 10.1152/jn.2002.87.6.2770

[B111] Seese R. R. Babayan A. H. Katz A. M. Cox C. D. Lauterborn J. C. Lynch G. (2012). LTP induction translocates cortactin at distant synapses in wild-type but not Fmr1 knock-out mice. J. Neurosci. 32, 7403–7413 10.1523/JNEUROSCI.0968-12.2012 22623686 PMC3365659

[B112] Semendeferi K. Armstrong E. Schleicher A. Zilles K. Van Hoesen G. W. (2001). Prefrontal cortex in humans and apes: a comparative study of area 10. Am. J. Phys. Anthropol. 114, 224–241 11241188 10.1002/1096-8644(200103)114:3<224::AID-AJPA1022>3.0.CO;2-I

[B113] Shieh P. B. Hu S.-C. Bobb K. Timmusk T. Ghosh A. (1998). Identification of a signaling pathway involved in calcium regulation of BDNF expression. Neuron 20, 727–740 10.1016/S0896-6273(00)81011-9 9581764

[B114] Shors T. J. Servatius R. J. Thompson R. F. Rogers G. Lynch G. (1995). Enhanced glutamatergic neurotransmission facilitates classical conditioning in the freely-moving rat. Neurosci. Lett. 186, 153–156 10.1016/0304-3940(95)11309-K 7777185

[B115] Simmons D. A. Mehta R. A. Lauterborn J. C. Gall C. M. Lynch G. (2011). Brief ampakine treatments slow the progression of Huntington's disease phenotypes in R6/2 mice. Neurobiol. Dis. 41, 436–444 10.1016/j.nbd.2010.10.015 20977939 PMC3014441

[B116] Simmons D. A. Rex C. S. Palmer L. Pandyarajan V. Fedulov V. Gall C. M. (2009). Up-regulating BDNF with an ampakine rescues synaptic plasticity and memory in Huntington's disease knockin mice. Proc. Natl. Acad. Sci. U.S.A. 106, 4906–4911 10.1073/pnas.0811228106 19264961 PMC2660722

[B117] Sirvio J. Larson J. Quach C. N. Rogers G. A. Lynch G. (1996). Effects of pharmacologically facilitating glutamatergic transmission in the trisynaptic intrahippocampal circuit. Neuroscience 74, 1025–1035 8895871 10.1016/0306-4522(96)00170-4

[B118] Staubli U. Chun D. Lynch G. (1998). Time-dependent reversal of long-term potentiation by an integrin antagonist. J. Neurosci. 18, 3460–3469 9547253 10.1523/JNEUROSCI.18-09-03460.1998 PMC6792671

[B119] Staubli U. Lynch G. (1987). Stable hippocampal long-term potentiation elicited by ‘theta’ pattern stimulation. Brain Res. 435, 227–234 10.1016/0006-8993(87)91605-2 3427453

[B120] Staubli U. Rogers G. Lynch G. (1994a). Facilitation of glutamate receptors enhances memory. Proc. Natl. Acad. Sci. U.S.A. 91, 777–781 10.1073/pnas.91.2.777 8290599 PMC43032

[B121] Staubli U. Perez Y. Xu F. Rogers G. Ingvar M. Stone-Elander S. (1994b). Centrally active modulators of glutamate (AMPA) receptors facilitate the induction of LTP *in vivo*. Proc. Natl. Acad. Sci. U.S.A. 91, 11158–11162 10.1073/pnas.91.23.11158 7972026 PMC45186

[B122] Staubli U. Vanderklish P. W. Lynch G. (1990). An inhibitor of integrin receptors blocks LTP. Behav. Neural Biol. 53, 1–5 10.1016/0163-1047(90)90712-F 2154174

[B123] Tong L. Prieto G. A. Kramar E. A. Smith E. D. Cribbs D. H. Lynch G. (2012). Brain-derived neurotrophic factor-dependent synaptic plasticity is suppressed by interleukin-1ß via p38 mitogen-activated protein kinase. J. Neurosci. 32, 17714–17724 10.1523/JNEUROSCI.1253-12.2012 23223292 PMC3687587

[B124] Turner D. C. Robbins T. W. Clark L. Aron A. R. Dowson J. Sahakian B. J. (2003). Cognitive enhancing effects of modafinil in healthy volunteers. Psychopharmacology (Berl.) 165, 260–269 12417966 10.1007/s00213-002-1250-8

[B125] van der Worp H. B. Howells D. W. Sena E. S. Porritt M. J. Rewell S. O'Collins V. (2010). Can animal models of disease reliably inform human studies. PLoS Med. 7:e1000245 10.1371/journal.pmed.1000245 20361020 PMC2846855

[B126] von der Brelie C. Waltereit R. Zhang L. Beck H. Kirschstein T. (2006). Impaired synaptic plasticity in a rat model of tuberous sclerosis. Eur. J. Neurosci. 23, 686–692 10.1111/j.1460-9568.2006.04594.x 16487150

[B127] Warren M. S. Bradley W. D. Gourley S. L. Lin Y. C. Simpson M. A. Reichardt L. F. (2012). Integrin beta1 signals through arg to regulate postnatal dendritic arborization, synapse density, and behavior. J. Neurosci. 32, 2824–2834 10.1523/JNEUROSCI.3942-11.2012 22357865 PMC3313657

[B128] Wickelgren W. A. (1974). Single-trace fragility theory of memory dynamics. Mem. Cogn. 2, 775–780 10.3758/BF03198154 24203753

[B129] Wiesner S. Legate K. R. Fassler R. (2005). Integrin-actin interactions. Cell Mol. Life Sci. 62, 1081–1099 10.1007/s00018-005-4522-8 15761669 PMC11139080

[B130] Xiao P. Bahr B. Staubli U. Vanderklish P. Lynch G. (1991). Evidence that matrix recognition contributes to stabilization but not induction of LTP. Neuroreport 2, 461–464 10.1097/00001756-199108000-00013 1912480

[B131] Zuccato C. Ciammola A. Rigamonti D. Leavitt B. R. Goffredo D. Conti L. (2001). Loss of huntingtin-mediated BDNF gene transcription in Huntington's disease. Science 293, 493–498 10.1126/science.1059581 11408619

